# Developing an Audit and Feedback Dashboard for Family Physicians: User-Centered Design Process

**DOI:** 10.2196/47718

**Published:** 2023-11-09

**Authors:** Jennifer Shuldiner, Tara Kiran, Payal Agarwal, Maryam Daneshvarfard, Kirsten Eldridge, Susie Kim, Michelle Greiver, Iffat Jokhio, Noah Ivers

**Affiliations:** 1 Women's College Hospital Toronto, ON Canada; 2 Department of Family and Community Medicine University of Toronto Toronto, ON Canada; 3 St Michael’s Hospital Unity Health Toronto Toronto, ON Canada; 4 MAP Centre for Urban Health Solutions St Michael’s Hospital Toronto, ON Canada; 5 Institute of Health Policy, Management and Evaluation University of Toronto Toronto, ON Canada; 6 Women’s College Academic Family Health Team Women’s College Hospital Toronto, ON Canada; 7 North York General Hospital Office of Research and Innovation Toronto, ON Canada; 8 Pivot Design Group Toronto, ON Canada

**Keywords:** audit and feedback, primary care, design, user-centered, design, audit, feedback, development, dashboard, family physician, clinical performance, implementation, users, primary care, care

## Abstract

**Background:**

Audit and feedback (A&F), the summary and provision of clinical performance data, is a common quality improvement strategy. Successful design and implementation of A&F—or any quality improvement strategy—should incorporate evidence-informed best practices as well as context-specific end user input.

**Objective:**

We used A&F theory and user-centered design to inform the development of a web-based primary care A&F dashboard. We describe the design process and how it influenced the design of the dashboard.

**Methods:**

Our design process included 3 phases: prototype development based on A&F theory and input from clinical improvement leaders; workshop with family physician quality improvement leaders to develop personas (ie, fictional users that represent an archetype character representative of our key users) and application of those personas to design decisions; and user-centered interviews with family physicians to learn about the physician’s reactions to the revised dashboard.

**Results:**

The team applied A&F best practices to the dashboard prototype. Personas were used to identify target groups with challenges and behaviors as a tool for informed design decision-making. Our workshop produced 3 user personas, Dr Skeptic, Frazzled Physician, and Eager Implementer, representing common users based on the team’s experience of A&F. Interviews were conducted to further validate findings from the persona workshop and found that (1) physicians were interested in how they compare with peers; however, if performance was above average, they were not motivated to improve even if gaps compared to other standards in their care remained; (2) burnout levels were high as physicians are trying to catch up on missed care during the pandemic and are therefore less motivated to act on the data; and (3) additional desired features included integration within the electronic medical record, and more up-to-date and accurate data.

**Conclusions:**

We found that carefully incorporating data from user interviews helped operationalize generic best practices for A&F to achieve an acceptable dashboard that could meet the needs and goals of physicians. We demonstrate such a design process in this paper. A&F dashboards should address physicians’ data skepticism, present data in a way that spurs action, and support physicians to have the time and capacity to engage in quality improvement work; the steps we followed may help those responsible for quality improvement strategy implementation achieve these aims.

## Introduction

Audit and feedback (A&F) involves delivering a summary of a recipient’s performance and is widely used as a quality improvement strategy across health settings to enable data-driven improvement [1]. Reporting metrics may include laboratory testing, adherence to clinical guidelines, patient experience data, disease-specific clinical quality measures, or prescribing.

Research has demonstrated that A&F has modest effects, with a Cochrane review demonstrating a 4.3% absolute improvement in health care professionals’ adherence to desired practices, such as recommended investigations or prescribing [1]. However, there was a large variation in effect size with some having an effect size of 16% while a quarter had a null or negative impact.

Evidence indicates that the design, usability, and method of delivery have a large impact on the effectiveness of A&F [1,2]. For A&F to lead to improvement, those getting the feedback must understand, accept, and act upon the results. However, clinicians might feel threatened rather than supported by top-down feedback and appropriately question whether the benefits to patient care rewards outweigh the efforts invested [3].

The design and delivery of A&F can be enhanced both through A&F theory and user-centered design methodology. A recent report from the US Agency for Healthcare Research and Quality [4] suggests that user-centered design can add value by ensuring that the end users’ perspectives are integrated into the design process [5]. User-centered design is an iterative and highly stakeholder-engaged process for generating products directly responsive to their intended contexts [6].

Our design aim was to produce a clinical dashboard for family physicians that would facilitate and encourage proactive preventative care from the family physician. However, in the context of inadequate health human resources and postpandemic burnout, we anticipated the challenges with the engagement of the family physician dashboard that was being developed. We hypothesized that combining A&F best practices with user-centered research into the design and implementation of A&F would address critical gaps that may inhibit the effectiveness of this quality improvement tool. In this paper, we describe the process of leveraging theory-based best practices in tandem with user-centered approaches to enhance the functionality, accessibility, and impact of a clinical dashboard for family physicians. We describe the process and outputs to inform others facing similar challenges when seeking to implement quality improvement strategies in primary care.

## Methods

### Overview

**Figure 1:**
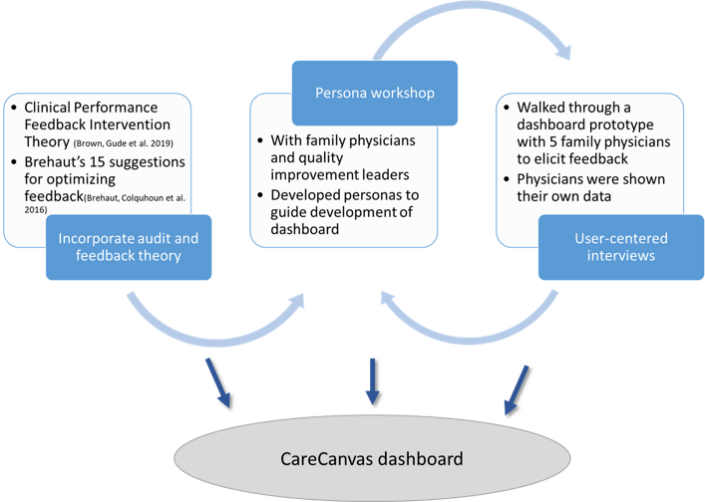
Design process of the audit and feedback CareCanvas dashboard for family physicians.

We engaged in an iterative multistep process combining A&F best practices with user-centered research, in the design and development of a web-based HTML dashboard for family physicians, CareCanvas. The process included (1) revisions to the prototype based on A&F theory; (2) a workshop with family physician quality improvement leaders to develop personas (ie, fictional characters that represent an archetype character); and (3) user-centered interviews with family physicians to learn about the physician’s reactions to the dashboard (Figure 1). We discuss the feedback we gathered in each of these 3 stages and how they influenced dashboard design. The research team worked with Pivot Design Group (Ian Chalmers, David Brennan, IJ) through this process and included consultation with a working group of primary care leaders, quality Improvement leaders, and researchers.

### CareCanvas

CareCanvas is a web-based HTML-based dashboard using electronic medical record (EMR) data. It leverages a secure researchable database comprised of deidentified patient records that can be reidentified at the practice level. The initial rollout included more than 15 quality-of-care measures built on existing data algorithms developed by the quality improvement program directors at the Department of Family and Community Medicine, University of Toronto. The directors collectively agreed to a set of quality improvement indicators that are meaningful and feasible to generate from available data. Currently, these indicators focus on hypertension, diabetes, and prescribing antibiotics, opioids, and other medications. The purpose of developing the dashboard was to support family physicians to use their data for learning and improvement, encourage proactive care, and help with catching up on missed care during the beginning of COVID-19. The initial prototype was ready in the fall of 2021 and the design process described in this paper spanned from the fall of 2021 to the summer of 2022.

### Prototype Development Based on A&F Theory and Input From Clinical Improvement Leaders

Fifteen indicators were chosen in a separate process for the dashboard based on the availability of EMR data, existing algorithms available to identify chronic conditions, and consultation with Quality Improvement Leads at the Department of Family and Community Medicine at the University of Toronto. The initial dashboard prototype was developed by a dually trained family doctor and engineer on the study team (Adam Cadotte).

Next, the team worked on updating the prototype by incorporating best practices from leading papers that summarize recommendations on the design of A&F [2,3,7]. Two A&F syntheses offer helpful insights. The first combines systematic review and expert interviews to summarize 15 practical ways to increase the impact of feedback [3]. The second synthesized 65 qualitative evaluations to produce a theory explaining what factors influence feedback success [2]. The team assessed its fit with suggestions, and then decisions on changes were made iteratively in consultation with the larger team and clinical quality leaders associated with the University of Toronto.

### Cocreation Workshop With Family Physician Quality Improvement Leaders to Develop Personas

We used user-centered design methods from design thinking, a “human-centered approach to innovation—anchored in understanding customer's needs, rapid prototyping, and generating creative ideas” [8]. We used these methods to gain a deeper, empathic understanding of the physicians using the dashboard. We conducted a workshop to develop personas that would guide our decision-making in developing the dashboard. The personas are fictional characters that represent an archetype personality. The personas guided the team in identifying physicians’ needs and wishes and enabled the team to engage and empathize during the design process.

The personas were first created by the research team by drawing upon theories [2], research [9,10], and personal experiences. The general details of the personas (eg, Dr Frazzled Physician or Dr Eager Implementer) were then presented to a group of family physicians who are part of the Quality Improvement Leads at the Department of Family and Community Medicine at the University of Toronto at a workshop for feedback. Next, physicians were split into groups where they discussed the goals, barriers, and what may help to overcome those barriers for each persona. Each session was recorded and had a notetaker. Following the workshop, recordings and notes were reviewed and summarized.

### User-Centered Interviews With Family Physicians

We recruited family physicians through clinical leads at participating sites. Recruitment was targeted and aimed to include a diverse group of physicians regarding gender, years in practice, and type of practice (community vs academic). We invited physicians to participate in a 1-time 60-minute interview to review their personalized dashboard prototype. The “think-aloud” method encouraged participants to share thoughts, reactions, likes, and dislikes as they went through the dashboard [11]. We also asked physicians clarifying questions and probed on the accuracy of the data and what they might do with a dashboard (Multimedia Appendix 1). The interviews were recorded and the study team reviewed the recordings and extracted data into the template to capture reflections and themes for each indicator. Next, the team reviewed the data extraction table for key themes that could inform design changes and also researchers’ observations of physicians’ nonverbal reactions and emotional responses. Following the 5 interviews, the team prepared a presentation for the larger team which met to discuss the problems identified during the user testing sessions and assess the severity of the issues and possible ways to address them in the context of the overall goal of the dashboard and best practices of A&F.

### Ethical Considerations

This initiative was formally reviewed by institutional authorities at Women’s College Hospital and was deemed not to require Research Ethics Board approval. It received approval from Women’s College Hospital Assessment Process for Quality Improvement Projects (#2021-0143-P).

## Results

### Prototype Development Based on A&F Theory and Input From Clinical Improvement Leaders

**Figure 2:**
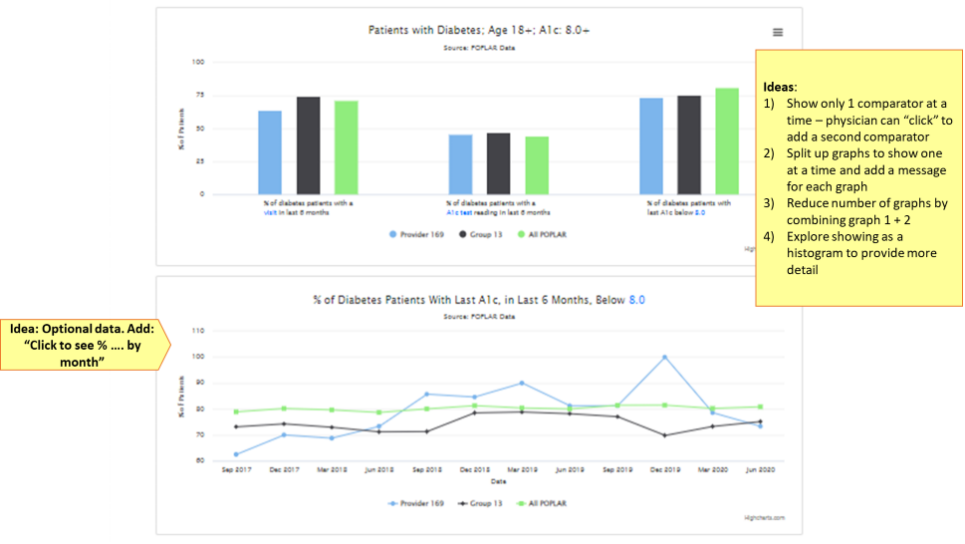
Original prototype for diabetes indicators. A_1c_: hemoglobin A_1c_.

The team assessed each indicator and suggested recommendations to ensure that the dashboard was adherent to the best practices of A&F (Figure 2). For example, the following recommendations were made regarding the diabetes indicator: (1) reduce cognitive load by allowing physicians to choose which comparator they want to see, (2) reduce cognitive load by presenting 1 indicator at a time in a given chart, (3) provide feedback in more than 1 way by adding a statement adjacent to the graph, (4) add action box to facilitate desired behaviors, and (5) ensure “download a list of patients who may require follow up” is easy to access to encourage the desired behavior.

### Cocreation Workshop With Family Physician Quality Improvement Leaders to Develop Personas

**Figure 3:**
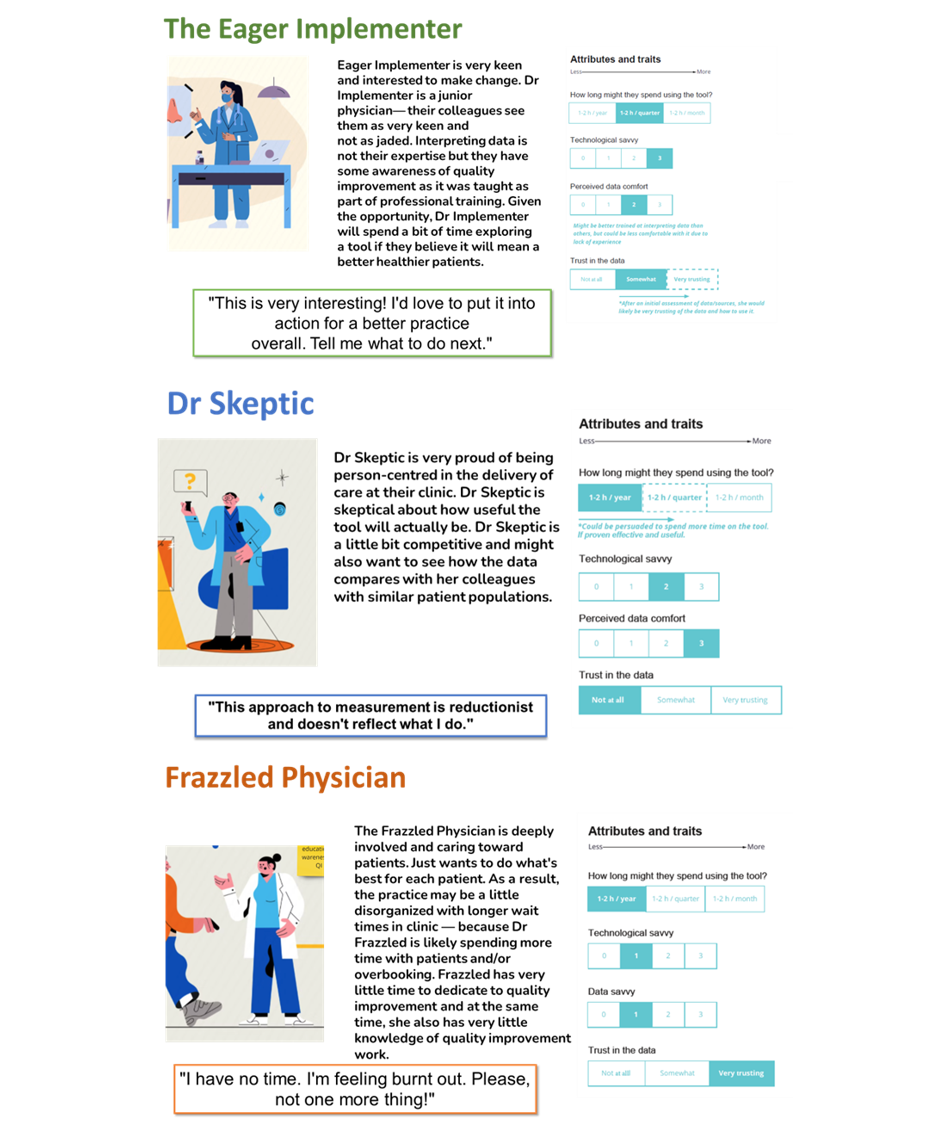
Priority personas developed and validated during the workshop.

The team along with the Pivot Design Group, developed personas based on the A&F literature [1,7,12-14] and their own experiences as family physicians and researchers of A&F [9,10,14-17]. In our workshop of 24 family physicians, Quality Improvement Leads at the Department of Family and Community Medicine at the University of Toronto, we sought input and validated the 3 personas we had developed: Dr Skeptic, Frazzled Physician, and The Eager Implementer (Figure 3). These 3 personas were selected because the team felt they were the most helpful caricatures of local family physicians to consider in the design and implementation of this A&F program. The personas were then validated and elucidated at the workshop where the physicians provided specific examples regarding their goals, pain points, and motivation for using A&F. See Multimedia Appendix 2 for an example of feedback provided in the workshop.

The first was Dr Skeptic, a physician who is very proud of delivering person-centered care at their clinic; however, they are also skeptical about how useful a dashboard will be. Dr Skeptic is a bit competitive and is interested to see how the data compare with colleagues with similar patient populations. Dr Skeptic might be persuaded to use the tool if a colleague has shared it, it was easy to use and understand, and they trusted the source of data and those sending it.

The Frazzled Physician is deeply involved and caring toward patients. This physician wants to do what is best for each patient. As a result, their practice may be disorganized and have longer wait times because Dr Frazzled is spending more time with patients and overbooking. Dr Frazzled has very little time to dedicate to quality improvement. They are comfortable with technology and with a little education on using the dashboard effectively, Dr Frazzled could make time to use the information. If they trust the dashboard they would say “If you gave me a list of patients to contact for XYZ reason, I would do it. Just tell me what to do.” They also need extra resources to help manage their time.

Dr Eager Implementer is very keen and interested in making changes. Dr Implementer is a junior physician. Their colleagues see them as very keen and not as jaded as some of the older physicians. Interpreting data is not their expertise, but they are tech-savvy. They have some awareness of quality improvement as it was taught in medical school. Given the opportunity, Dr Implementer will likely spend a bit of time exploring a dashboard if prompted and given the right opportunity.

The team used these personas for the remainder of the design process to guide our design decisions. Some of the common aspects of the personas that the team considered were their lack of time and burnout, wish to provide quality clinical care, and desire to keep up with their peers. An effort was made to ensure things were clear and simple because it was recognized that data and tech savviness would vary. The team tried to incorporate each persona into their decisions so that the dashboard would suit the persona’s needs, goals, and motivations. Their roadblocks and frustrations and what might motivate them to use the dashboard were considered (Table 1).

**Table 1:** Examples of how the team used personas to address design decisions during CareCanvas development.

Design question	Personas considered	Design decision
Do we include a “target”?	Dr Skeptic would question the “target” causing them to disengage with the dashboard. Dr Frazzled might feel that the dashboard was being judgmental and punitive.	Did not include targets for indicators.
Where do we provide information regarding indicators and data?	Dr Skeptic may wish to see the precise definitions for several indicators but Dr Frazzled and Dr Eager implementer might not need this data and might get distracted.	Include a “more info” that is faint but accessible near every indicator.
How do we describe patients that need follow-up care?	Dr Frazzled and Dr Skeptic might disengage from the dashboard if it seems punitive and triggering and it is not a place of positive support.	Switch from “Patients at risk” to “Patients that may benefit from follow-up”
Which action cards should appear in the beginning of the dashboard?	Dr Frazzled and Dr Skeptic would be interested in action cards that are straightforward for follow-up. Ensure limited number of action cards so as not to overwhelm the physicians.	Prioritizing what to highlight for follow-up and limiting to 3 action cards per page.
How do we organize resources in the dashboard?	All personas would benefit from organization of resources. Dr Eager implementer might want to send list of patient resources to their patients.	Split into patient and physician resources. Include only 3-4 items per section.
Prevalence graphs—should we include comparators?	All personas would not benefit from comparisons as it would not enable them to compare the quality of care they provide to their peers.	Did not include comparators for certain indicators (eg, opioids).
What cut-offs should be used for clinical indicators (eg, whether patients are below a specific A1c or BP value)?	Dr Frazzled likely prefers simplicity while Dr Skeptic may have strong views about the optimal cut-off that should be used.	Include toggles for clinical values where there may be reasonable disagreement but maintain a default view for simplicity.

### User-Centered Interviews With Family Physicians

We then conducted 5 user-centered interviews with family physicians (Table 2; the summary of results can be found in Table 3). Physicians had a range of visual preferences. For example, some physicians preferred to view their data in graphs, while others wished to see a declarative statement summarizing key points. There were also differences in what types of comparators were preferred, for example, region, clinic, and provincial. Consistent preferences included the wish to see raw numbers alongside percentages (ie, 20% of patients have high blood pressure corresponding to 35 patients) and the desire to avoid cognitive overload when physicians were presented with too much data at 1 time.

**Table 2:** Characteristics of physicians who participated in user-centered interviews.

Characteristic		Values
**Gender, n (%)**		
	Male	2 (40)
	Female	3 (60)
Years practicing medicine, mean (SD)		25 (13)
Number of patients, mean (SD)		1050 (560)
**Type of practice, n (%)**		
	Family Health Team	5 (100)

**Table 3:** Supporting quotes to learnings from user-centered interviews.

Themes from interviews	Supporting quotes	Implications for dashboard
Meaningful values	“The data does not seem relevant to my practice because of the glycemic and blood pressure target...if I’m not getting all of my patients under 8.5 I’m not doing a good job as a clinician” (physician 4).	Indicators were made to be customizable so that physicians could control cut-off point for values.
Desire for actionable data	“I don’t know how useful this is to me. This information doesn’t change how I practice” (physician 3).	Data that were deemed unactionable were removed from the dashboard. For example, comparison of a physician’s rate of opioid prescriptions to other physicians because it is not clear whether peer data represent a desirable target.
Data accurate and timely	“Dashboard needs to be current -1-3 months old is fine” (physician 4).	Efforts were made to ensure timely data. We added a time stamp in the dashboard so physicians can see the timeliness of the data.
Comparators and trends	“This is probably very important comparing yourself to your group and colleagues and prescription is always important to try to minimize, and if you see you are trending up I need to do something with this” (physician 1).	We added various comparator options with a button to enable choice regarding which comparator to view. We also included data on trends over time for each indicator.
Integration of workflow	“I want to get specific lists, and also if the list is not linked to the EMR I don’t know how many more steps I need to take...I have to type...it needs to be efficient and the way I suggest [linked to chart] is the most efficient way” (physician 5).	Download list were made easily accessible throughout the dashboard. The team is planning to develop instructions and a video to help physicians download the patient list and integrate it within their EMR^a^.
Burnt out and focused on catch-up care	“I don’t have time to look at data to make myself better. At this juncture I see this as a project to better myself...we are playing so much defense...We are playing damage control...3 years ago it would have been different” (physician 3).	We framed the dashboard as a tool to help physicians catch up on care that was missed during the pandemic. The team avoided negative statements or using “targets.” Efforts are ongoing to minimize work on behalf of the physician to access the dashboard and develop support to help with using the dashboard to improve patient care.
Comparing oneself to the mean	“It’s reassuring when you see similar patterns in the group when the result is not so good” (physician 1). “Would look at this to see if they are doing whatever others are doing and if the numbers are dramatically out of norm then would certainly try to correct” (physician 2).	Action cards were included at the top of the dashboard highlighting patients that required follow-up. This was meant to encourage physicians to download the patient list and follow-up with patients.

^a^EMR: electronic medical record.

Physicians voiced concerns regarding the perceived value of the dashboard. Many physicians already receive A&F products and, therefore, they wanted to know what the “value-add” was with CareCanvas. They expressed a desire for a dashboard that they could easily validate with their EMR. They also wanted their dashboard to include data that would trigger specific actionable tasks.

Physicians also expressed the desire for data that were current and accurate, and that the dashboard should be easily integrated within their workflow, for example, it was crucial to them that it should be integrated into their EMR to allow for easy access and facilitate following up with patients that required action.

General feedback on clinical topics included the desire to customize the indicators so that values were meaningful to them. For example, physicians wanted to decide what glycemic control value was presented in their dashboard. They also did not wish to see data that were perceived as unactionable. The data in the dashboard were seen as a request, and therefore, if it was not clear what the “ask” was, they described being frustrated. Finally, data on trends were highly desirable and crucial for them to assess if the given indicator should prompt clinical action (ie, if they were trending in the undesired direction, that gave them an incentive to act).

Physicians were very interested in how they compared to the average and would often dismiss feedback indicating gaps in care if their peers were experiencing similar results (eg, accepting if a certain proportion of their patients with diabetes had not had a blood pressure check in the last year if it was consistent with the average among all physicians). Finally, an overarching theme from physicians was that using and acting on a dashboard was not the top priority for them as they were feeling burnt out and were busy catching up on missed care from COVID-19.

## Discussion

### Principal Findings

Our paper outlines an A&F dashboard design process that harmonizes theory-based best practices and local users’ goals, preferences, problems of interest, and information needs. The method guided the selection of measures, development of functionality, and data visualization; we found it crucial to draw upon both best practices of A&F and user feedback when developing the dashboard. Our key learnings indicate that a successful design and implementation of an A&F dashboard for family physicians should address physicians’ data skepticism, present data in a way that spurs action, and support physicians to have the time and capacity to engage in quality improvement work. In describing our design process for the dashboard, we focus on issues that are likely to be generalizable to other teams developing theory-informed A&F materials.

It is common for the design of A&F to use behavioral theory [18]. However, it is less common for user-centered methods to be incorporated [19-21]. There is increasing evidence of the importance of using user-centered methods to improve user experience in health care interventions [12,22,23]. Implementing any quality improvement project necessitates an understanding of context [24], and we found that using user-centered methods was a thorough and beneficial way to understand and incorporate these perspectives into the design and implementation of the dashboard.

Some teams have used user interviews and multiple cycles of iterations in the design of an A&F [19,20]. Others have used a mix of behavioral theory and cocreation workshops to create emails to promote the use of A&F [16]. Methodologies differ; however, there is an underlying consensus that user-centered approaches optimize the functionality and uptake of interventions. Similarly, we found that applying A&F best practices in a context that is not well-suited can compromise its effectiveness and turn away users. Our development process sought to create a dashboard that balanced A&F theory with the data we were collecting from physician users and our process met 10 out of 11 criteria for user-centeredness (Multimedia Appendix 3), as assessed by the User-Centered Design 11-item measure [25].

Our process revealed tensions between user-centered design and A&F theory, thereby highlighting the necessity of using a user-centered approach. During the user-centered interviews, a variety of barriers were identified that we attempted to address in the design, many of which would not have come up in A&F theory. For example, the need to address overwhelming feelings of burnout after the challenges of the COVID-19 pandemic, and the sense that physicians and their clinics were working at capacity. We addressed these findings by ensuring the dashboard was framed positively, even if this meant compromising best practices according to the A&F literature. For example, A&F literature recommends using a “target” or “best performing” to push physicians to act, as often the average physician has room for improvement but might not be motivated if they see they are performing like their peers. However, we decided not to include a “target” performance measure as it could be demoralizing for physicians, especially in the context of COVID-19. The team also decided to forgo using a summative declarative statement adjacent to graphs to avoid perceived judgment and critique. In these design decisions, the team sought to balance A&F best practices while being mindful of physician wellness and capacity and our goal of engaging physicians in improvement work over the long-term.

Using personas in the design process enabled the group to make design decisions while considering the goals, motivations, and barriers of physicians in mind. As the team was developing personas, some were not a priority as they either represented a small number of physicians or were not personas likely to engage with an A&F dashboard. The team selected a few priority personas that were used throughout the design process so we could aim to accommodate all varying needs of the personas as decisions were being made.

Through our user-centered process, there were learnings regarding implementing this methodology. Notably, we learned the value of showing users their personal data during a feedback session. This elicited a stronger reaction to the data, a more critical eye, and we were able to witness interaction of feedback in real time.

There were also challenges in embedding user-centered methodology into the design process. Extensive engagement with users can be time-intensive and costly. Our group had to juggle the importance of user engagement with deadlines that were important to stakeholders. Issues of sampling and recruitment are crucial, and we are aware the findings can depend on who is recruited for user testing. Our team tried to recruit physicians who resembled a “typical” user that represented users more broadly and practiced in different types of practice (academic vs nonacademic) and varying age groups. This work was done in an urban academic center and based in primary care which may limit its external generalizability to other locations and specialties of medicine. The process we used, however, to collect insights relevant to the local context is entirely transferable.

### Conclusions

There is a need to embed user-centered research into the design and implementation of A&F to address critical gaps that are inhibiting the effectiveness of this quality improvement tool. We leveraged methods from user-centered design methodology to harmonize A&F theory and context and found that user engagement led to crucial design changes. User-centered methodology allowed the team to embed users more deeply in the process through personas and user testing. These methods elicited concerns that if left unaddressed, could have limited its uptake and let our team design a dashboard that maximizes usability and usefulness.

## Appendix

Multimedia Appendix 1 Interview guide for physician interviews.

Multimedia Appendix 2 Physician workshop breakout group for Dr. Skeptic.

Multimedia Appendix 3 11-Item measure of user- and human-centered design for personal health tools (UCD-11).

## Abbreviations

A&F: audit and feedback
EMR: electronic medical record
