# Supplementary material for: Developing an Audit and Feedback Dashboard for Family Physicians: User-Centered Design Process
Source: JMIR Hum Factors. 2023 Nov 9;10:e47718. doi: 10.2196/47718 (PMC10667970; doi:10.2196/47718)
Supplement: Multimedia Appendix 1 [file humanfactors_v10i1e47718_app1.docx]

1. Demographic and physician characteristics
   1. How old are you?
   2. How long have you been practicing?
   3. What type of practice do you have? Is it team-based? (i.e. FHT…)
   4. Do you use or look at any other dashboards? My practice report? i4c dashboard?
   5. How many patients do you have?
   6. How many days a week do you practice?
   7. Who else works in the office with you?
      1. How many other physicians?
      2. Nurses? Pharmacists?
      3. Social Workers?
      4. Other support staff?

Ok Great. Thank you for all of that information. Let’s start looking at your dashboard.

**Review dashboard for Feedback: *Highlight that we are assessing the tool not you!* Ask participants to talk out loud their thoughts as they review.**

As you have heard, we are designing dashboard for family physicians in the University of Toronto Practice-Based Research Network. We would like to go through the dashboard with you to hear your initial reactions, opinions and thoughts to whatever you see. Make sure you verbalize what you are looking at and how it makes you feel. This will help us to redesign the dashboard to ensure that it is as helpful as possible to you, your colleagues, and your patients.

**B. Introduction**

First I am going to ask you to look at this X section and tell me what you make of it.

1. Can you share what are you thinking as you first look at the dashboard?
2. What is the first thing that draws your attention?
3. Any initial thoughts?
4. **Characteristics of patients**

Take a look at this section and tell me what you make of it.

1. What do you think someone might learn from learn from this information?
2. Do these numbers seem accurate to you?
3. Was is it useful?
4. Was there anything unclear?
5. Anything you would have wanted to know?
6. Any other thoughts regarding this section?
7. **Hypertension**

Take a look at this section and tell me what you make of it.

1. What do you think someone might learn from the data on [above category]? Feedback]
   1. Any other thoughts?
   2. Does this data seem relevant to your practice? [interaction and perception] How would you rate that on a scale of 1-5? Why?
   3. Does this data seem accurate? [Verification] How would you rate that on a scale of 1-5? Why?
   4. If a physician had issues with accuracy or did not trust the information, how do you think the dashboard/report might help them overcome this?
2. Your data was presented with comparators- any thoughts on this? Did this influence the way you saw you data? How so? [Perception - Benchmarking Trend] (probe on the usefulness of each comparator)
3. What did you think of the recommended actions? Any other suggestions? [Intention, Behaviour]
4. What did you think of the toolbox?
5. After seeing this data, has it impacted how you think of your practice regarding this issue? [interaction and perception] (probe to find out what influenced)
6. Is there anything you think you might want to do with this data you have just seen regarding [above category] ? [Intention, Behaviour] (probe to find out what influenced)

**H. Dashboard’s design**

1. Now that we have looked at the whole dashboard – I am curious to hear about your thoughts regarding design: [usability]
   - Layout?
   - How was the order?
   - Was it clear? How would you rate that on a scale of 1-5? Why?
   - Were you able to navigate easily? How would you rate that on a scale of 1-5? Why?
   - Colour?
   - Is there anything that you would change?

**I. Overall comments and debrief**

Now that you we have gone over the whole dashboard…

1. What did you think? [interaction and perception]
2. Was it useful? Relevant? [interaction and perception]
3. Did you think is accurately represented your clinical practice [acceptance]
4. Is there anything you would like to do with the data you have just learnt? [Intention]
   1. How do you think you might to do that? [goal setting]
5. Is there any other data you would like to see included? If you had access to a dashboard like this, what kind of information might be most useful to you in your practice? (Please explain.)
6. What did you think of the length? [Perception]
7. Is this something you would like to receive? [Perception]
8. When do you think you would want to receive this dashboard? If you had access to a dashboard like this, when might you use it? (Prompts: What time of day? Between patients? At the end of the day? At home?) And how often might you use it? *— Aiming to understand how it might best fit in their day-to-day workflow*
   1. How often?
9. Is there anything else you would like to receive with the dashboard to help? Any other supprts? eg peer coaching, lunch and learn on a topic etc.
   1. What would make it easier for someone to use/understand this tool more effectively?
   2. Have you had a CPSO QI review- was this helpful?
10. Are there any wider system changes at your clinic you think may help with using this dashboard to provide better patient care? [Clinical performance improvement]
11. If you could change/add/remove ONE thing about the tool to make it more useful and relevant to others like you, what would you change/add/remove and why?
12. What, if anything, would make you want to recommend this dashboard to a colleague? *(Prompts: a specific feature, a data set, or some other part of the experience?)*
13. Any other thoughts? Comments? Suggestions?

Let participant know that a gift card will be emailed to them and thank them for attending the interview.
