# Supplementary figures and images for: Developing an Audit and Feedback Dashboard for Family Physicians: User-Centered Design Process
Source: JMIR Hum Factors. 2023 Nov 9;10:e47718. doi: 10.2196/47718 (PMC10667970; doi:10.2196/47718)

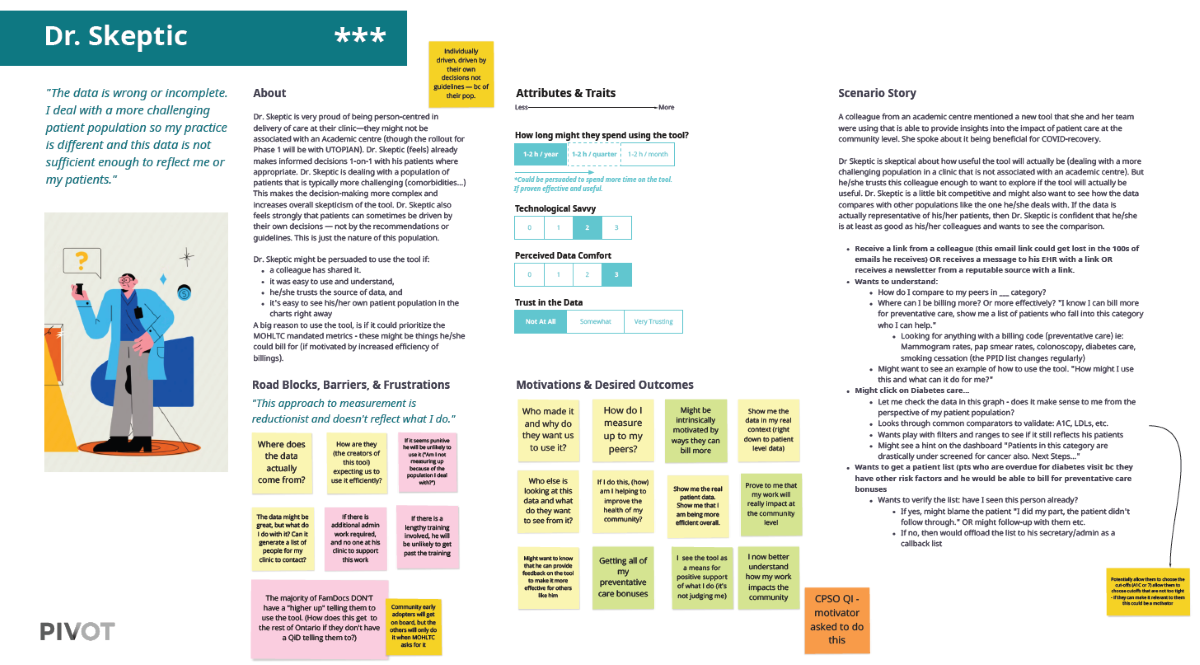

Supplement: Multimedia Appendix 2 [file humanfactors_v10i1e47718_app2.png]
