# Supplementary material for: Developing an Audit and Feedback Dashboard for Family Physicians: User-Centered Design Process
Source: JMIR Hum Factors. 2023 Nov 9;10:e47718. doi: 10.2196/47718 (PMC10667970; doi:10.2196/47718)
Supplement: Multimedia Appendix 3 [file humanfactors_v10i1e47718_app3.docx]

| Items | Explanations and examples | Yes/No |
| --- | --- | --- |
| 1. Were potential end users (eg, patients, caregivers, family and friends, surrogates) involved in any steps to help understand users (eg, who they are, in what context might they use the tool) and their needs? | Such steps could include various forms of user research, including formal or informal needs assessment, focus groups, surveys, contextual inquiry, ethnographic observation of existing practices, literature review in which users were involved in appraising and interpreting existing literature, development of user groups, personas, user profiles, tasks, or scenarios, or other activities | Yes |
| 2. Were potential end users involved in any steps of designing, developing, and/or refining a prototype? | Such steps could include storyboarding, reviewing the draft design or content before starting to develop the tool, and designing, developing, or refining a prototype | Yes |
| 3. Were potential end users involved in any steps intended to evaluate prototypes or a final version of the tool? | Such steps could include feasibility testing, usability testing with iterative prototypes, pilot testing, a randomized controlled trial of a final version of the tool, or other activities | Yes |
| 4. Were potential end users asked their opinions of the tool in any way? | For example, they might be asked to voice their opinions in a focus group, interview, survey, or through other methods | Yes |
| 5. Were potential end users observed using the tool in any way? | For example, they might be observed in a think-aloud study, cognitive interviews, through passive observation, logfiles, or other methods | Yes |
| 6. Did the development process have 3 or more iterative cycles? | The definition of a cycle is that the team developed something and showed it to at least one person outside the team before making changes; each new cycle leads to a version of the tool that has been revised in some small or large way | No |
| 7. Were changes between iterative cycles explicitly reported in any way? | For example, the team might have explicitly reported them in a peer-reviewed paper or in a technical report. In the case of rapid prototyping, such reporting could be, for example, a list of design decisions made and the rationale for the decisions | Yes |
| 8. Were health professionals asked their opinion of the tool at any point? | Health professionals could be any relevant professionals, including physicians, nurses, allied health providers, etc. These professionals are not members of the research team. They provide care to people who are likely users of the tool. Asking for their opinion means simply asking for feedback, in contrast to, for example, observing their interaction with the tool or assessing the impact of the tool on health professionals’ behavior | Yes |
| 9. Were health professionals consulted before the first prototype was developed? | Consulting before the first prototype means consulting prior to developing anything. This may include a variety of consultation method | Yes |
| 10. Were health professionals consulted between initial and final prototypes? | Consulting between initial and final prototypes means some initial design of the tool was already created when consulting with health professionals | Yes |
| 11. Was an expert panel involved? | An expert panel is typically an advisory panel composed of experts in areas relevant to the tool if such experts are not already present on the research team (eg, plain language experts, accessibility experts, designers, engineers, industrial designers, digital security experts, etc). These experts may be health professionals but not health professionals who would provide direct care to end user |  |
